# Prosthetic joint infection eradication outcomes for DAIR (debridement, antibiotics, and implant retention) procedure according to culture etiology

**DOI:** 10.1371/journal.pone.0356624

**Published:** 2026-08-25

**Authors:** Alexandra N. Sheldon, Douglas J. Chonko, Alexander H. Fischbach, Kenan Alzouhayli, Sina Ramtin, Kathryn Fideler, Roberto Gonzalez, Nathaniel A. Bates

**Affiliations:** 1 Department of Orthopaedics, The Ohio State University Wexner Medical Center, Columbus, Ohio, United States of America; 2 College of Medicine, The Ohio State University Wexner Medical Center, Columbus, Ohio, United States of America; 3 Division of Orthopedic Surgery, Mount Sinai Medical Center, Aventura, Florida, United States of America; Laurentian University, CANADA

## Abstract

**Aims:**

Prosthetic joint infection (PJI) is a rare but devastating complication following total hip and knee arthroplasty. While two-stage revision remains the gold standard, debridement, antibiotics, and implant retention (DAIR) offers a less morbid alternative for select patients. The aim of this study was to assess how microbiologic etiology affects DAIR success, with emphasis on fungal, antibiotic-resistant, and culture-negative PJIs, and to identify pathogen-specific predictors that may guide treatment decisions.

**Methods:**

A single-center retrospective review was conducted of 173 culture-positive and culture-negative PJIs treated with DAIR between January 2016 and June 2025. Inclusion criteria followed modified Musculoskeletal Infection Society (MSIS) guidelines. Demographics, comorbidities, and microbiologic data were collected. Treatment success was defined as infection eradication without recurrence or need for further surgical intervention, per Delphi consensus. Logistic regression analyses were performed to evaluate associations between treatment success and microbiologic characteristics, as well as between fungal PJI and patient comorbidities.

**Results:**

Overall, infection eradication was achieved in 66.5% of cases. Fungal pathogens were significantly associated with DAIR failure compared to non-fungal organisms (Odds Ratio [OR] 0.06, 95% Confidence Interval [CI] 0.011–0.31, P = 0.001). Culture-negative infections were nearly four times more likely to achieve eradication (OR 3.91, 95% CI 1.43–10.69, P = 0.008). Antibiotic-resistant and gram-positive pathogens were associated with lower success, whereas polymicrobial infections did not differ significantly from single-organism infections (P value = 0.92). Fungal PJIs comprised 8% of cases and exhibited a higher comorbidity burden (median Charlson Comorbidity Index [CCI] 4 vs 2, P = 0.0496).

**Conclusions:**

Fungal organisms and antibiotic-resistant pathogens were associated with lower rates of infection eradication, underscoring the importance of pathogen-specific risk stratification when considering DAIR. These findings suggest that alternative surgical strategies or heightened surveillance may be warranted in patients with high-risk organisms.

## Introduction

Total knee arthroplasty is a well-known approach for patients with debilitating, end-stage osteoarthritis, with over 1 million total knee and total hip replacements performed each year [1]. Both the incidence and prevalence of these procedures are predicted to increase in the coming years as our population ages.

Prosthetic joint infections (PJIs) are rare complications of total knee arthroplasty (TKA) and total hip arthroplasty (THA), affecting approximately 1–2% of all total hips and knees [2,3]. However, while uncommon, PJIs are devastating complications for the arthroplasty surgeon and patient. The goal of PJI treatment is to effectively manage the infection while preserving joint function and minimizing additional complications such as loss of time at work, arthrofibrosis, spread of the infection, antibiotic resistance, pressure ulcers, amputation, Deep vein thrombosis (DVT), and death. Two-stage revision surgery is often considered the gold standard approach to PJIs, however the DAIR procedure—debridement, antibiotics, and implant retention—has been advocated as a viable alternative [4]. Implant retention (without recurrent infection) is an ideal treatment course as DAIR procedures reduce patient morbidity and associated health care costs [3,5]. DAIR proponents also cite improved implant function and equivalent hardware longevity when compared with two-stage revisions [3,6]. These comparisons support DAIR as an established treatment option for appropriately selected patients with PJI. These comparisons make DAIR a worthwhile emerging treatment option for eligible patients. Good candidates for DAIR are often identified in the immediate postoperative period of the index surgery and consist of individuals who are otherwise relatively healthy, with a previously well-functioning implant and good soft tissue coverage [4,7].

Growing evidence indicates the decision to retain an implant may be pathogen-dependent [8]. DAIR is safely recommended in an immunocompetent host with low virulence organisms, such as coagulase-negative streptococcus, because they do not as readily form biofilms [4,8]. Conversely, an implant should not be retained in MRSA cases, as studies have shown high failure rates with this pathogen [9–11]. However, literature remains inconclusive regarding the role of microbiology as a predictor for infection eradication for other less prominent organisms, including fungal pathogens, which only encompass ~1–2% of all PJIs [12,13]. Due to this relatively low rate of incidence, fungal PJIs remain understudied in the literature, leaving a pertinent gap in knowledge regarding optimal treatment pathways relative to multi-stage and DAIR procedures.

The purpose of this study was to determine which pathogen etiology is more likely to lead to success in clearing PJIs via the DAIR procedure. We hypothesized that rates of infection eradication would differ based on organism etiology. With the knowledge gained from this study, approaches to PJI treatment can be more confidently driven by microbiologic diagnosis to enhance likelihood of successful patient outcomes.

## Methods

This retrospective study was reviewed and approved by the Institutional Review Board (IRB) (IRB #2021H0358). Following approval, we conducted a single-center retrospective chart review of patients who came for treatment of PJI that followed either total hip arthroplasty (THA) or total knee arthroplasty (TKA), between 01/01/2016–06/20/2025. The data were accessed between 05/01/2025− 12/01/2025. Patients were identified according to the International Classification of Diseases-10 [ICD-10] code diagnosis of prosthetic joint infection. For this study, patients were only included if they underwent DAIR as the primary procedure to address their PJI. Patients were excluded if they underwent incision and debridement (I&D), single stage, explant only, or explant and spacer as the primary procedure to address their PJI. Patients were also excluded if their index TKA or THA was for a primary malignancy or bone sarcoma.

Criteria established by the Musculoskeletal Infection Society (MSIS) in 2011 and further refined by Parvizi et al in 2018 are largely accepted to define PJI [14]. A version of these criteria modified to fit the testing parameters available at our center was used to determine positive classification of PJI in the present patient population. Specifically, synovial C-reactive protein (CRP) is not collected at our institution and, as such, was eliminated from the standard model of determination. Cases were initially identified using ICD-10 codes. Following chart review, only patients meeting the modified MSIS criteria for PJI were included in the final study cohort. Table 1 provides a detailed breakdown of the process used to classify presence of PJI within a THA or TKA patient. In addition, our institution holds all aerobic and anaerobic cultures specimens for at least 14 days after collection. All fungal cultures are held for 28 days, and all acid-fast culture specimens are held for 42 days. The Microbiology Department flags all concerns for contaminants, and these contaminated specimens were not included as culture positive results. Antimicrobial regimens were left to the discretion of the treating physician and infectious disease team. Culture negative PJIs were, at a minimum, addressed with broad spectrum antibiotic coverage for both gram positive and gram-negative organisms.

**Table 1:** Modified Musculoskeletal Infection Society (MSIS) Criteria for the diagnosis and definition of a periprosthetic joint infection (PJI).

Major Criteria			
Two positive cultures of the same organism			
Sinus tract with evidence of communication to the joint or visualization of the prosthesis			
**Decision:** Presence of **one major criterion = Infected**			
Preoperative diagnosis	**Minor Criteria**		Score
	Serum	Elevated CPR (>1.0mg/dL in Chronic or >10mg/dL in Acute)	2
		Elevated ESR (>30mm/h)	1
	Synovial	Elevated synovial WBC count (>3,000 cells/uL)	3
		Positive alpha-defensin (Synovasure) (send-out positive/negative lab)	3
		Elevated synovial PMN % (>80%)	2
**Decision based on total score:** The cumulative score from the minor criteria was used to classify infection status. A total score of **≥6 points was considered infected**, **2–5 points possibly infected**, and **0–1 points not infected**. Operative criteria can be used to fulfill the PJI definition for patients with inconclusive minor criteria.			
Intraoperative Diagnosis		Inconclusive pre-operative score or dry tap	Score
		Positive histology	3
		Positive purulence	3
		Single positive culture	2
**Decision based on total score:** When preoperative evaluation was inconclusive or a dry tap occurred, intraoperative findings were incorporated into the scoring system. A total score of **≥6 points was classified as infected**, **4–5 points as possibly infected** ^a^, and **≤3 points as not infected**.			

Cases classified as **possibly infected** were often referred for additional molecular diagnostic testing, such as **next-generation sequencing (NGS)** [14].

CRP, C-reactive protein; ESR, erythrocyte sedimentation rate; PMN, polymorphonuclear; WBC, white blood cell.

Data was systematically abstracted from the electronic medical record into the PJI database by the research team. Demographic data included sex, age at PJI diagnosis, laterality, infection chronicity, total hip or total knee arthroplasty, body mass index (BMI), smoking status, medical comorbidities, MSIS criteria (including serum and synovial laboratory values). Patient culture results were also abstracted and included culture negative status, fungal pathogen status, polymicrobial status, antibiotic resistant status, gram-negative status, and gram-positive status. A single outcome variable was abstracted as outcome success, which was defined as the successful eradication of infection as stratified by infection etiology. Fungal infections consisted predominantly of Candida species. Due to the limited number of infections within individual pathogen species, organisms were grouped into broader microbiological categories for analysis. Antibiotic-resistant organisms were defined as pathogens demonstrating resistance to standard antimicrobial therapy. Success was defined as (1) patient alive (2) meeting criteria based on the Delphi international multidisciplinary consensus: infection eradication characterized by a healed wound without drainage, fistula, or pain and no infection recurrence; no occurrence of periprosthetic joint infection-related mortality (e.g., sepsis, necrotizing fasciitis); and no subsequent planned surgical intervention for infection [15]. Patients were considered failures if were placed on chronic suppressive antibiotics, had other signs/symptoms of persistent infection, were deceased, or needed an amputation. Postoperative follow-up from the most recent surgery was available for 159 patients, with a median follow-up duration of 227 days (IQR 71–748 days). Treatment success was determined using all available follow-up documented within the electronic medical record.

Abstracted data were statistically analyzed using Stata Statistical Software (StataCorp LLC, College Station, TX, USA). Logistic regression analyses were performed to evaluate associations between treatment success and microbiological characteristics, as well as between fungal periprosthetic joint infection (PJI) and patient comorbidities. Continuous variables were examined for normality using skewness and kurtosis and summarized as mean ± standard deviation or median (interquartile range) as appropriate. Statistical significance was defined as a p-value < 0.05 and an odds ratio with a 95% confidence interval.

## Results

### Demographics

A total of 173 PJIs were identified for inclusion in this study for pathogen investigation. 36% of these PJIs were infected THAs and 64% were infected TKAs. Of the cohort, 58% of the patients were female and 42% were male. The median time from index surgery to periprosthetic joint infection (PJI) was 537 days (Interquartile Range [IQR] 35–2428 days). The average age of PJI diagnosis was 63 years (Standard Deviation [SD] = 10.7 years). The average BMI of our cohort was 33.8 (SD = 7.8) and only 10% of our patients had a healthy weight range BMI (18.5–24.9) (Table 2).

**Table 2:** Baseline Demographic, Clinical, and Microbiological Characteristics of Patients Undergoing DAIR for PJI (n = 173).

Variable		
Age at PJI, years	Mean ± SD	62.6 ± 10.7
Body Mass Index (BMI)	Median (IQR)	33.5 (28.6–39.0)
Time from index surgery to PJI, days	Median (IQR)	537 (35–2428)
		**n (%)**
Sex	Female	101 (58.4)
	Male	72 (41.6)
Arthroplasty type	TKA	111 (64.2)
	THA	62 (35.8)
Smoking status	Never	81 (46.8)
	Former	73 (42.2)
	Current	19 (11.0)
Treatment success	Yes	115 (66.5)
	No	58 (33.5)
Culture results	Bacterial	121 (69.9%)121 (69.9%)
	Culture-negative	37 (21.4%)37 (21.4%)
	Fungal	14 (8.1%)14 (8.1%)
	Acid-fast bacilli	1 (0.6%)1 (0.6%)
Culture characteristics	Polymicrobial	51 (29.5)
	Antibiotic-resistant organism	28 (16.2)
Gram-positive organisms	*Staphylococcus aureus (MSSA)*	38 (22.0)
	*Staphylococcus aureus (MRSA)*	21 (12.1)
	*S. epidermidis (MSSE)*	8 (4.6)
	*S. epidermidis (MRSE)*	8 (4.6)
	Coagulase-negative Staphylococcus	10 (5.8)
	*Cutibacterium acnes*	19 (11.0)
	*Enterococcus spp.*	12 (6.9)
	VRE (Enterococcus spp.)	5 (2.9)
	*Corynebacterium spp.*	12 (6.9)
	Group A Streptococcus	2 (1.2)
	Group B Streptococcus	10 (5.8)
	Group G Streptococcus	2 (1.2)
	Viridans group Streptococcus	4 (2.3)
	Other Streptococcus	1 (0.6)
	Other Gram-positive	11 (6.4)
Gram-negative organisms	*Enterobacter spp.*	8 (4.6)
	*Pseudomonas aeruginosa*	11 (6.4)
	Other Gram-negative	22 (12.7)
Other organisms	Fungal	14 (8.1)
	Acid-fast bacilli	1 (0.6)
	Culture-negative	36 (20.8)

DAIR, Debridement, Antibiotics, and Implant Retention, TKA, total knee arthroplasty; THA, total hip arthroplasty; PJI, periprosthetic joint infection; MSSA, methicillin-sensitive Staphylococcus aureus; MRSA, methicillin-resistant Staphylococcus aureus; MSSE, methicillin-sensitive Staphylococcus epidermidis; MRSE, methicillin-resistant Staphylococcus epidermidis; VRE, vancomycin-resistant Enterococcus; IQR, interquartile range.

Values are presented as number (percentage) unless otherwise indicated

### Success by organism

Overall, infection was successfully eradicated with the DAIR procedure in 66.5% of all PJIs. Polymicrobial pathogens did not exhibit a significant difference in successful eradication compared to single microbial pathogens (Odds Ratio [OR] 0.62, 95% Confidence Interval [95% CI] 0.32, 1.23, *P*-value = 0.170). Accounting for polymicrobial pathogens as a confounding variable in the logistic regression analysis, fungal pathogens were independently significantly more likely to fail to eradicate infection compared to non-fungal pathogens (OR 0.06, 95% CI 0.011, 0.31, *P*-value = 0.001). Culture negative PJIs were significantly more likely to result in successful infection eradication compared to culture positive PJIs (OR 3.9, 95% CI 1.43, 10.69, *P*-value = 0.008). Antibiotic resistant pathogens were significantly more likely to fail to eradicate infection compared to non-resistant pathogens (OR 0.31, 95% CI 0.13, 0.70, *P*-value = 0.005). Accounting for polymicrobial pathogens as a confounding variable in the logistic regression analysis, Gram positive organisms were significantly less likely to result in a successful eradication compared to organisms that were not gram positive (OR 0.33, 95% CI 0.15, 0.73, *P*-value = 0.007), however, Gram-negative organisms did not exhibit a significant difference in successful eradication compared to organisms that were not gram negative (OR 0.59, 95% CI 0.21, 1.68, *P*-value = 0.32) (Table 3).

**Table 3:** Logistic Regression Analyses of Factors Associated with Treatment Success After DAIR for PJI (n = 173).

Model/ Predictor	Odds Ratio (95% CI)	Std. Error	p-value	Pseudo R²
**Poly-microbial infection**	0.62 (0.32–1.23)	0.22	0.17	0.008
**Polymicrobial + Fungal infection**				0.082
Polymicrobial	1.21 (0.54–2.74)	0.5	0.65	
Fungal	0.06 (0.01–0.31)	0.05	**0.001**	
**Culture-negative PJI**	3.91 (1.43–10.7)	2.01	**0.008**	0.04
**Antibiotic-resistant organism**	0.31 (0.13–0.70)	0.13	**0.005**	0.036
**Polymicrobial + Antibiotic-resistant**				0.037
Polymicrobial	0.82 (0.39–1.69)	0.3	0.59	
Antibiotic-resistant	0.33 (0.14–0.79)	0.15	**0.012**	
**Gram-status model**				0.048
Gram-positive infection	0.33 (0.15–0.73)	0.13	**0.007**	
Gram-negative infection	0.59 (0.21–1.68)	0.31	0.32	
Polymicrobial	0.96 (0.46–2.02)	0.36	0.92	

DAIR, debridement, antibiotics, and implant retention; PJI, periprosthetic joint infection; CI, confidence interval.

Values in bold indicate statistical significance at p < 0.05

### Fungal

Given the high rate of fungal PJIs (Table 2) and the higher likelihood of treatment failure, comorbidities were independently assessed within this cohort of patients (Table 4). Peripheral vascular disease was the only comorbidity significantly more prevalent in the fungal cohort compared with non-fungal etiologies (OR 5.92, 95% CI 1.34–26.1; P = 0.019). However, the median of the Charlson Comorbidity Index (CCI) in the fungal PJI group was 4 (IQR = 2–6) compared to 2 (IQR = 1–3) for the non-fungal cohort, which is statistically different (*P* = 0.0496). Of the 14 fungal PJI cases, 13 (93%) were polymicrobial with a superimposed bacterial infection.

**Table 4:** Bivariate Logistic Regression Analyses of Risk Factors Associated with Fungal PJI (n = 173).

Predictor	Odds Ratio (95% CI)	Std. Error	p-value	Pseudo R²
**Liver disease**	1.96 (0.22–17)	2.2	0.55	0.003
**Number of complications**	1.14 (0.97–1.4)	0.1	0.11	0.023
**Malignancy**	1.87 (0.38–9.3)	1.5	0.44	0.005
**Peripheral vascular disease (PVD)**	**5.92 (1.34–26)**	4.5	**0.019**	0.046
**Type 2 diabetes mellitus (T2DM)**	2.47 (0.81–7.6)	1.4	0.11	0.024
**Substance abuse**	1.45 (0.17–13)	1.6	0.73	0.001

PJI = periprosthetic joint infection; CCI = Charlson Comorbidity Index; PVD = peripheral vascular disease; T2DM = type 2 diabetes mellitus; CI = confidence interval.

Values in bold indicate statistical significance (p < 0.05)

## Discussion

Microbiology plays a crucial role in the diagnosis, management, and treatment of prosthetic joint infections (PJIs) [2]. Accurate identification of the causative pathogen from blood, tissue, and synovial samples allows for tailored antibiotic treatment and appropriate planning for further surgical intervention, which ultimately improves outcomes [2,16]. The objective of this study was to assess how microbiologic etiology influences infection eradication following DAIR. Our findings demonstrated that overall infection eradication was achieved in 66.5% of all PJIs, which aligns with reported rates in the literature ranging from 60% to 90% [2,4,13]. Given that our institution is a tertiary referral center, this may explain why results fall on the lower end of the eradication statistic, because many of our patients arrive from external care facilities after initial intervention has already failed. Specifically, fungal pathogens were significantly more likely to fail eradication compared to non-fungal organisms, whereas culture-negative infections were associated with higher odds of success. Although most fungal infections were polymicrobial, their poorer outcomes indicate that the fungal component itself contributes to DAIR failure rather than the polymicrobial nature of infection [13]. In contrast, polymicrobial infections alone did not show a significant difference in success compared to single-organism infections. These observations underscore the prognostic importance of microbiologic diagnosis in PJI management and highlight the challenges posed by fungal and resistant pathogens in achieving durable infection control [17,18].

### Fungal

Our results indicated that the incidence of fungal PJIs in our cohort was 8%, which is much higher than the literature reports of only 1–2% of all PJI cases [12,13,19,20]. Though fungal PJIs are rare, they have increasing incidences in specific patient populations with underlying decreased immunity from immunosuppressive diseases or drugs, overuse or misuse of antibiotics, diabetes, or intravenous drug and substance abuse that leave the host susceptible [13,21–23]. Our center is a referral center for PJI patients, and thus, the likelihood of fungal PJI may increase in our patient population. While we only found peripheral vascular disease to occur with fungal PJIs more commonly, the burden of average total comorbid conditions was twice as high as in fungal patients compared to nonfungal patients. Other studies have identified several comorbidities and risk factors for fungal PJIs; however, the biggest risk factor is prior antimicrobial therapy within 3-months of infection [12,24]. Because so many o patients in this investigation arrived from outside hospitals, medical records were not sufficient to determine what antimicrobials, if any, had been prescribed prior to infection.

The present data also indicated that fungal PJIs had worse success with infection eradication when compared to bacterial infections. Much of the pathogenesis of fungal infections explains this modulating effect. Fungal pathogens are known to form robust biofilms that adhere to prosthetic implants [25]. These biofilms protect the fungus from the host immune response and antimicrobials. Fungi also have more significant tissue penetration, which may require additional aggressive surgical interventions [26,27]. Because of this, in the setting of fungal PJI, DAIR may not be the procedure of choice as a retained implant seems to increase the risk for failure to eliminate the infectious source [7]. Many patients with fungal PJIs also can have superimposed bacteria. In our study, more than 90% of patients also had at least one additional culture positive bacteria. This further complicates management and treatment tolerability. The interaction of bacterial superinfection on fungal outcomes was not explored due to low sample sizes but should be investigated in future studies.

Patients with fungal PJIs often have delayed diagnosis. This is primarily due to their indolent clinical presentation and difficulty isolating fungal organisms from cultures. Fungal infections trigger a less pronounced inflammatory response compared to bacterial infections, delaying the immune system’s recognition and response. Ultimately, patients do not look as sick and have less alarming laboratory findings (inflammatory markers such as CRP and synovial cell counts) [12]. However, fungus is extremely difficult to isolate and takes more time than bacteria to identify [16]. In this time, medical and surgical management is often initiated. DAIR is commonly performed in the early postoperative period; thus, a patient may undergo surgical management before fungal cultures have come back positive. While the temporality of culture collection and procedure were not assessed in this study, it is a safe recommendation that if fungus is identified preoperatively, the surgeon should consider alternative treatment options outside of DAIR [7].

Prior use of antibiotics is just one of the many challenges to culture yields. Isolating fungal pathogens demands enriched media and prolonged incubation, often exceeding four weeks [13]. Given these limitations, molecular diagnostics have become increasingly important. PCR and next-generation sequencing (NGS) now provide rapid turnaround within a week and detect up to 90% of pathogens, especially in culture-negative cases [13,16]. Microbiology departments must have the capacity, both physically and financially, to store these cultures and run appropriate diagnostic tests. Large academic centers are more capable of handling the thousands of samples that must be tested and stored. Funded institutions also have access the most up-to-date technologies and research-driven practices. Thus, fungal PJI rates at our institution may be higher due to sheer advances in ability to diagnose infections appropriately and efficiently.

### Culture negative

We showed that culture negative PJIs were approximately 4 times more likely to lead to treatment success in infection eradication. This may be due to several factors. Often, the reason for culture negative PJIs is due to prior use of antibiotics. Empiric coverage for PJIs includes a broad spectrum of antibiotics that cover a wide range of pathogens that are the most common and most virulent among PJIs [28]. A culture negative result may suggest that the organism was susceptible to the antibiotic and is no longer detectable in tissue, blood, or synovial fluid samples. Many studies suggest that the underlying organisms of culture negative PJIs are in fact less virulent, and the organisms count is lower, suggesting these may actually be a positive prognostic factor for patients [29]. Furthermore, the use of broad-spectrum antibiotics allows for coverage of multiple pathogenic organisms, such as in the case of polymicrobial infections. However, when an organism is isolated early on, treatment is immediately narrowed to more targeted antibiotics, which may then not cover organisms that are present but take longer to isolate. Thus, the use of broader spectrum antibiotics in culture negative PJIs may allow for better coverage in polymicrobial circumstances [28]. Overall, culture negative PJIs can actually be a reassuring prognostic factor for patients, though the search for a microbial diagnosis should not be discontinued.

Overall, 21% of PJIs were classified as culture negative. While reported literature varies, some cite rates as high as 45% [30]. The lower rates in our study may again be attributable to our institution. Our academic center has funding to afford most state-of-the-art diagnostic tests and treatments. With the most advanced technology and experts at our disposal, high diagnostic yield likely accounts for our relatively lower prevalence of culture negative PJIs.

### Limitations

Our study is limited by its retrospective nature. Follow-up duration was variable across patients, with a median postoperative follow-up of 227 days. As a result, late recurrences may not have been captured in all cases, potentially leading to underestimation of treatment failure rates. Further, follow-up periods were not unified across all patients. Future study should regulate follow-up periods to determine treatment outcome relative to designated time frames. A further limitation exists in the collection of culture specimens as collection and result timeframes were not regulated in relation to surgical and antimicrobial treatment. Thus, relative incubation period was not assessed for each subject. An additional limitation is the potential for incomplete capture of care received at outside institutions, which may have resulted in underreporting of subsequent procedures, complications, or infection recurrence not documented within our health system.

## Conclusion

Fungal organisms and antibiotic-resistant pathogens were associated with lower rates of infection eradication, underscoring the importance of pathogen-specific risk stratification when considering DAIR. These findings suggest that alternative surgical strategies or heightened surveillance may be warranted in patients with high-risk organisms.

## References

[1] Shichman I, Roof M, Askew N, Nherera L, Rozell JC, Seyler TM. Projections and epidemiology of primary hip and knee arthroplasty in Medicare patients to 2040-2060. JBJS Open Access. 2023;8. doi: 10.2106/JBJS.OA.22.00112 PMC9974080 36864906

[2] Zimmerli W, Trampuz A, Ochsner PE. Prosthetic-Joint Infections. N Engl J Med. 2004;351:1645–54. doi: 10.1056/NEJMra040181 15483283

[3] Grammatopoulos G, Bolduc M-E, Atkins BL, Kendrick BJL, McLardy-Smith P, Murray DW, et al. Functional outcome of debridement, antibiotics and implant retention in periprosthetic joint infection involving the hip: a case-control study. Bone Joint J. 2017;99-B(5):614–22. doi: 10.1302/0301-620X.99B5.BJJ-2016-0562.R2 28455470

[4] Qasim SN, Swann A, Ashford R. The DAIR (debridement, antibiotics and implant retention) procedure for infected total knee replacement - a literature review. SICOT J. 2017;3:2. doi: 10.1051/sicotj/2016038 28074774 PMC5225833

[5] Antonios JK, Bozic KJ, Clarke HD, Spangehl MJ, Bingham JS, Schwartz AJ. Cost-effectiveness of Single vs Double Debridement and Implant Retention for Acute Periprosthetic Joint Infections in Total Knee Arthroplasty: A Markov Model. Arthroplast Today. 2021;11:187–95. doi: 10.1016/j.artd.2021.08.009 34660864 PMC8502838

[6] Kurtz SM, Ong KL, Lau E, Bozic KJ, Berry D, Parvizi J. Prosthetic joint infection risk after TKA in the Medicare population. Clin Orthop Relat Res. 2010;468(1):52–6. doi: 10.1007/s11999-009-1013-5 19669386 PMC2795807

[7] Vaz K, Taylor A, Kendrick B, Alvand A. A guide to debridement, antibiotics, and implant retention. Ann Jt. 2022;7:5. doi: 10.21037/aoj-20-89 38529146 PMC10929291

[8] Zürcher-Pfund L, Uçkay I, Legout L, Gamulin A, Vaudaux P, Peter R. Pathogen-driven decision for implant retention in the management of infected total knee prostheses. Int Orthop. 2013;37(8):1471–5. doi: 10.1007/s00264-013-1923-4 23695880 PMC3728396

[9] Ferry T, Uçkay I, Vaudaux P, François P, Schrenzel J, Harbarth S, et al. Risk factors for treatment failure in orthopedic device-related methicillin-resistant Staphylococcus aureus infection. Eur J Clin Microbiol Infect Dis. 2010;29(2):171–80. doi: 10.1007/s10096-009-0837-y 19946789

[10] Triantafyllopoulos GK, Poultsides LA, Zhang W, Sculco PK, Ma Y, Sculco TP. Periprosthetic knee infections treated with irrigation and debridement: outcomes and preoperative predictive factors. J Arthroplasty. 2015;30(4):649–57. doi: 10.1016/j.arth.2014.10.026 25466169

[11] Bradbury T, Fehring TK, Taunton M, Hanssen A, Azzam K, Parvizi J, et al. The fate of acute methicillin-resistant Staphylococcus aureus periprosthetic knee infections treated by open debridement and retention of components. J Arthroplasty. 2009;24(6 Suppl):101–4. doi: 10.1016/j.arth.2009.04.028 19553077

[12] Riaz T, Tande AJ, Steed LL, Demos HA, Salgado CD, Osmon DR, et al. Risk Factors for Fungal Prosthetic Joint Infection. J Bone Jt Infect. 2020;5(2):76–81. doi: 10.7150/jbji.40402 32454521 PMC7242408

[13] Chisari E, Lin F, Fei J, Parvizi J. Fungal periprosthetic joint infection: Rare but challenging problem. Chin J Traumatol. 2022;25(2):63–6. doi: 10.1016/j.cjtee.2021.12.006 35031203 PMC9039431

[14] Parvizi J, Tan TL, Goswami K, Higuera C, Della Valle C, Chen AF, et al. The 2018 Definition of Periprosthetic Hip and Knee Infection: An Evidence-Based and Validated Criteria. J Arthroplasty. 2018;33(5):1309-1314.e2. doi: 10.1016/j.arth.2018.02.078 29551303

[15] Diaz-Ledezma C, Higuera CA, Parvizi J. Success after treatment of periprosthetic joint infection: a Delphi-based international multidisciplinary consensus. Clin Orthop Relat Res. 2013;471(7):2374–82. doi: 10.1007/s11999-013-2866-1 23440616 PMC3676607

[16] Portillo ME, Sancho I. Advances in the Microbiological Diagnosis of Prosthetic Joint Infections. Diagnostics (Basel). 2023;13(4):809. doi: 10.3390/diagnostics13040809 36832297 PMC9954824

[17] Tande AJ, Patel R. Prosthetic Joint Infection. Clin Microbiol Rev. 2014;27:302–45. doi: 10.1128/CMR.00111-13 24696437 PMC3993098

[18] Baumgärtner T, Bdeir M, Dally F-J, Gravius S, Hai AAE, Assaf E, et al. Rifampin-resistant periprosthetic joint infections are associated with worse functional outcome in both acute and chronic infection types. Diagn Microbiol Infect Dis. 2024;110(2):116447. doi: 10.1016/j.diagmicrobio.2024.116447 39111108

[19] Kurmis AP. Eradicating Fungal Periprosthetic TKA “Super-infection”: Review of the Contemporary Literature and Consideration of Antibiotic-Impregnated Dissolving Calcium Sulfate Beads as a Novel PJI Treatment Adjunct. Arthroplast Today. 2021;8:163–70. doi: 10.1016/j.artd.2021.02.009 33855143 PMC8024748

[20] Gomez-Urena EO, Tande AJ, Osmon DR, Berbari EF. Diagnosis of Prosthetic Joint Infection. Infectious Disease Clinics of North America. 2017;31(2):219–35. doi: 10.1016/j.idc.2017.01.008 28483043

[21] Ueng SWN, Lee C-Y, Hu C, Hsieh P-H, Chang Y. What Is the Success of Treatment of Hip and Knee Candidal Periprosthetic Joint Infection?. Clinical Orthopaedics & Related Research. 2013;471(9):3002–9. doi: 10.1007/s11999-013-3007-6 23633184 PMC3734391

[22] Brown TS, Petis SM, Osmon DR, Mabry TM, Berry DJ, Hanssen AD, et al. Periprosthetic Joint Infection With Fungal Pathogens. J Arthroplasty. 2018;33(8):2605–12. doi: 10.1016/j.arth.2018.03.003 29636249

[23] Belden K, Cao L, Chen J, Deng T, Fu J, Guan H, et al. Hip and knee section, fungal periprosthetic joint infection, diagnosis and treatment: Proceedings of international consensus on orthopedic infections. J Arthroplasty. 2019;34:S387–91. doi: 10.1016/j.arth.2018.09.023 30343967

[24] Azzam K, Parvizi J, Jungkind D, Hanssen A, Fehring T, Springer B, et al. Microbiological, clinical, and surgical features of fungal prosthetic joint infections: a multi-institutional experience. J Bone Joint Surg Am. 2009;91 Suppl 6:142–9. doi: 10.2106/JBJS.I.00574 19884422

[25] McConoughey SJ, Howlin R, Granger JF, Manring MM, Calhoun JH, Shirtliff M, et al. Biofilms in periprosthetic orthopedic infections. Future Microbiol. 2014;9(8):987–1007. doi: 10.2217/fmb.14.64 25302955 PMC4407677

[26] Kojic EM, Darouiche RO. Candida infections of medical devices. Clin Microbiol Rev. 2004;17(2):255–67. doi: 10.1128/CMR.17.2.255-267.2004 15084500 PMC387407

[27] Zimmerli W, Sendi P. Orthopaedic biofilm infections. APMIS. 2017;125(4):353–64. doi: 10.1111/apm.12687 28407423

[28] Le Vavasseur B, Zeller V. Antibiotic therapy for prosthetic joint infections: an overview. Antibiotics (Basel). 2022;11(4):486. doi: 10.3390/antibiotics11040486 35453237 PMC9025623

[29] Watanabe S, Kobayashi N, Tomoyama A, Choe H, Yamazaki E, Inaba Y. Clinical characteristics and risk factors for culture-negative periprosthetic joint infections. J Orthop Surg Res. 2021;16(1):292. doi: 10.1186/s13018-021-02450-1 33941220 PMC8091510

[30] Tan TL, Kheir MM, Shohat N, Tan DD, Kheir M, Chen C. Culture-negative periprosthetic joint infection: an update on what to expect. JBJS Open Access. 2018;3:e0060. doi: 10.2106/JBJS.OA.17.00060 PMC6242327 30533595

